# Grb2 interacts with necrosome components and is involved in rasfonin-induced necroptosis

**DOI:** 10.1038/s41420-022-01106-1

**Published:** 2022-07-13

**Authors:** Bolin Hou, Haiwen Huang, Yueqian Li, Jingnan Liang, Zhijun Xi, Xuejun Jiang, Ling Liu, Erwei Li

**Affiliations:** 1 State Key Laboratory of Mycology, Institute of Microbiology, Chinese Academy of Sciences, Beijing, 100101 China; 2 CAS Key Laboratory of Microbial Physiological and Metabolic Engineering, Institute of Microbiology, Chinese Academy of Sciences, Beijing, 100101 China; 3 Department of Urology, Peking University First Hospital, Beijing, 100034 China; 4 School of Pharmacy and State Key Laboratory of Applied Organic Chemistry, Lanzhou University, Lanzhou, 730000 China; 5 Institutional Center for Shared Technologies and Facilities, Institute of Microbiology, Chinese Academy of Sciences, Beijing, 100101 China

**Keywords:** Macroautophagy, Necroptosis

## Abstract

The underlying mechanism by which growth factor receptor-bound protein 2 (Grb2) regulates necroptosis remains unexplored. In the present study, we found that rasfonin, a fungal natural product and an activator of necroptosis, enhanced Grb2 binding to receptor-interacting serine/threonine kinase 1 (RIP1), which plays a critical role in regulating programmed necrosis. Moreover, we observed that SQSTM/p62 (p62), a protein that can form necrosomes with RIP1, increased its interaction with Grb2 upon rasfonin challenge. Although it has been used as an activator of autophagy in our previous study, here we found that a high dose of rasfonin was able to inhibit autophagic process. Inhibition of RIP1 either chemically or genetically reversed the inhibition of rasfonin on autophagy, whereas knockdown of Grb2 markedly reduced rasfonin-induced necrosis. Additionally, we found that the compound failed to upregulate the expression of RIP1 in Grb2-deprived cells. In summary, our data revealed that Grb2 actively participated in rasfonin-induced necroptosis by interacting with the components of necrosome and mediating their expression.

## Introduction

Growth factor receptor bound protein 2 (Grb2), an epidermal growth factor receptor (EGFR) binding protein [1], is involved in tyrosine kinase regulated signal transduction [2, 3]. Grb2 has been discovered to form complexes with a range of cellular proteins and o have roles in cell growth, cell proliferation, metabolism, embryogenesis, and cancer cell differentiation [4, 5]. Despite its well-known role in connecting tyrosine kinase to activation of the Ras pathway [6], Grb2 has also been demonstrated to be linked to programmed cell death signaling [7]. Grb3-3, an alternative splicing form of Grb2 transcripts lacking the functional SH2 domain, has been shown to convey a signal causing cells to undergo apoptosis [1], while suppression of Grb2 expression has been shown to attenuate palmitic acid-induced apoptosis in HepG2 cells [8]. In addition to interaction with phosphotyrosine via its SH2 domain, Grb2 is also associated with tumor necrosis factor α (TNF-α) receptor I through one of its SH3 domains, which results in c-Raf-1 activation [9]. Importantly, Hitomi et al. (2008) discovered the adapter protein is necessary for both zVAD.fmk- and TNF-α-induced necroptosis [10].

Necroptosis belongs to one type of programmed cell death (PCD) that is the most well-studied form of programmed necrosis, triggered by several mechanisms [11]. Upon stimulation with TNF-α, necrosomes are formed by serine/threonine receptor-interacting protein 3 (RIPK3 or RIP3) in complex with RIPK1 (or RIP1), FADD, and caspase-8. Subsequently, mixed lineage kinase domain-like protein (MLKL) is recruited to necrosome complex, accompanied by MLKL phosphorylation via RIPK3 that induces necroptosis by mediating plasma membrane lysis [10, 12, 13]. However, in TNF-related apoptosis-inducing ligand (TRAIL)-activated necroptosis, multifunction protein p62/SQSTM1 [14] controls necrosome assembly by recruiting RIP1 [15].

Rasfonin, a natural product derived from the fermentation of *Talaromyces sp*. 3656-A1 [16], is named for its ability to inhibit the minor G protein Ras. In a recent work, we discovered that rasfonin triggered apoptosis and necrosis, as well as macroautophagy (hereafter called autophagy), which is a degrading process for maintaining homeostasis by catabolizing organelles and proteins to produce nutrients and macromolecular precursors [17, 18]. Generally, autophagy occurs at a basal level in nearly all eukaryotic cells, yet autophagy can lead to cell death when it is overactivated, resulting in a process called autophagic cell death [19]. Unlike the connection between autophagy and apoptosis, little is known about how autophagy regulates programmed necrosis.

Based on our previous investigation, we conducted this study to reveal the mechanism of action of rasfonin-induced necrosis. Here, we revealed that rasfonin could either promote or inhibit the autophagic process in a concentration-dependent manner. High dose of the rasfonin induced necroptosis, while suppressing autophagy. However, knockdown of RIP1 reversed its inhibitory effect on autophagy. Moreover, we found that Grb2 interacts with both RIP1 and p62 to regulate necroptosis, suggesting that Grb2 and its upstream EGFR are likely to be involved in switching between autophagy and programmed necrosis.

## Results

### High concentration of rasfonin inhibited autophagy and induced necroptosis

**Fig. 1:**
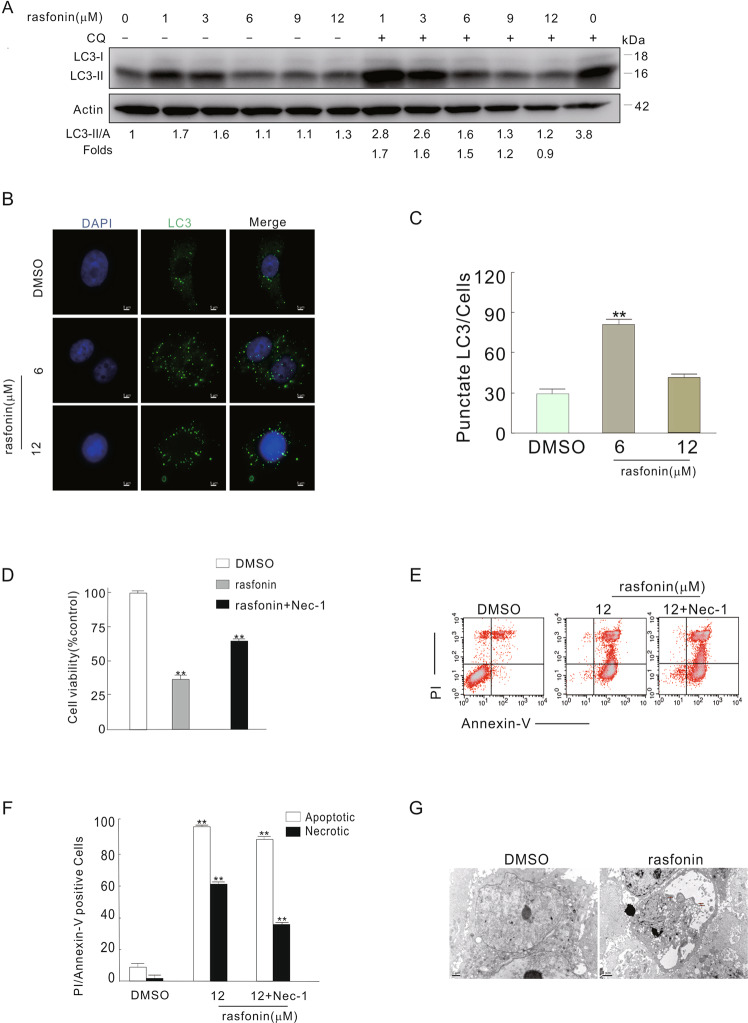
High concentration of rasfonin inhibited autophagy and induced necroptosis. **A** ACHN cells were treated with rasfonin (0–12) μM for 4 h, the cells were lysed and subjected to immunoblotting with the antibodies indicated. Actin was used as a loading control. **B,** **C** ACHN cells were split onto coverslips, cultured overnight, following rasfonin (6 or 12 μM) for 6 h. the cells were fixed with 4% paraformaldehyde, immunostained with anti-LC3 antibody and visualized by fluorescence microscopy The number of the punctate LC3 in each cell was counted, and at least 50 cells were included for each group. Data representing the mean ± S.D. were shown in graph. **D** ACHN cells were treated with rasfonin (12 μM) in the presence or absence of necrostatin 1 (Nec-1, 30 μM) for 12 h; cell viability was analyzed by MTS assay as described in Materials and Methods. **E**, **F** ACHN cells were treated with rasfonin (12 μM) alone or together with Nec-1 (30 μM) for 6 h, the induced apoptosis and necrosis were determined by flow cytometry. Apoptotic: AV-positive and PI-negative; necrotic: PI-positive. The data are presented as mean ± S.D. from three independent experiments. The double asterisk denotes the group is statistically different from the control groups (*P* < 0.01). **G** Electron microscopy was performed in ACHN cells following treatment of rasfonin (12 μM) for 6 h as described in “Materials and methods”. Arrow: vesiculation of cytoplasmic organelle; Double Arrows: rupture of the plasma membrane. Similar experiments were repeated at least three times.

In our previous study, we observed that rasfonin activated the autophagic process concurrent with the induction of programmed cell death [17]. To determine the type of rasfonin-activated programmed cell death, we employed various concentrations of the compound in the present study. Interestingly, we found that, while rasfonin promoted autophagic process until the 9 μM concentration, the compound was able to inhibit autophagy at a dose of 12 μM, as chloroquine (CQ), which is often used in detecting autophagic flux [18], failed to accumulate LC3-II at this concentration (Fig. 1A). By using fluorescence microscopy, we observed that rasfonin at a concentration of 12 μM induced much less punctate GFP-LC3 staining than rasfonin at a dose of 6 μM (Fig. 1B, C), whereas transmission electron microscopy (TEM) demonstrated that rasfonin (12 μM) induced an obvious decline in membrane vacuoles compared with 6 μM rasfonin (Supplement Fig. 1A, B). The aforementioned results indicated that rasfonin could either promote or suppress autophagy depending on the concentration used.

Allosteric inhibitor of RIP1, Nec-1 has been widely utilized as a tool to explore the molecular mechanism of programmed necrosis [20–22]. In this study, we observed that Nec-1 markedly reduced rasfonin-induced cell viability loss (Fig. 1D), whereas flow cytometry data demonstrated that the inhibitor markedly suppressed necrosis in rasfonin-treated cells (Fig. 1E, F), suggesting that rasfonin is able to activate necroptosis in ACHN cells. Additionally, TEM imaging revealed that rasfonin treatment could induce a typical necrotic cell death morphology [23], such as extensive vesiculation of cytoplasmic organelles and rupture of the plasma membrane (Fig. 1G). Moreover, phosphorylated H2AX (γH2AX), a sensitive marker for DNA damage and a regulator of necrotic PCD [5, 24], was markedly upregulated upon rasfonin challenge (Supplement Fig. 1C).

### Rasfonin failed to inhibit autophagy in RIP1-deprived cells

**Fig. 2:**
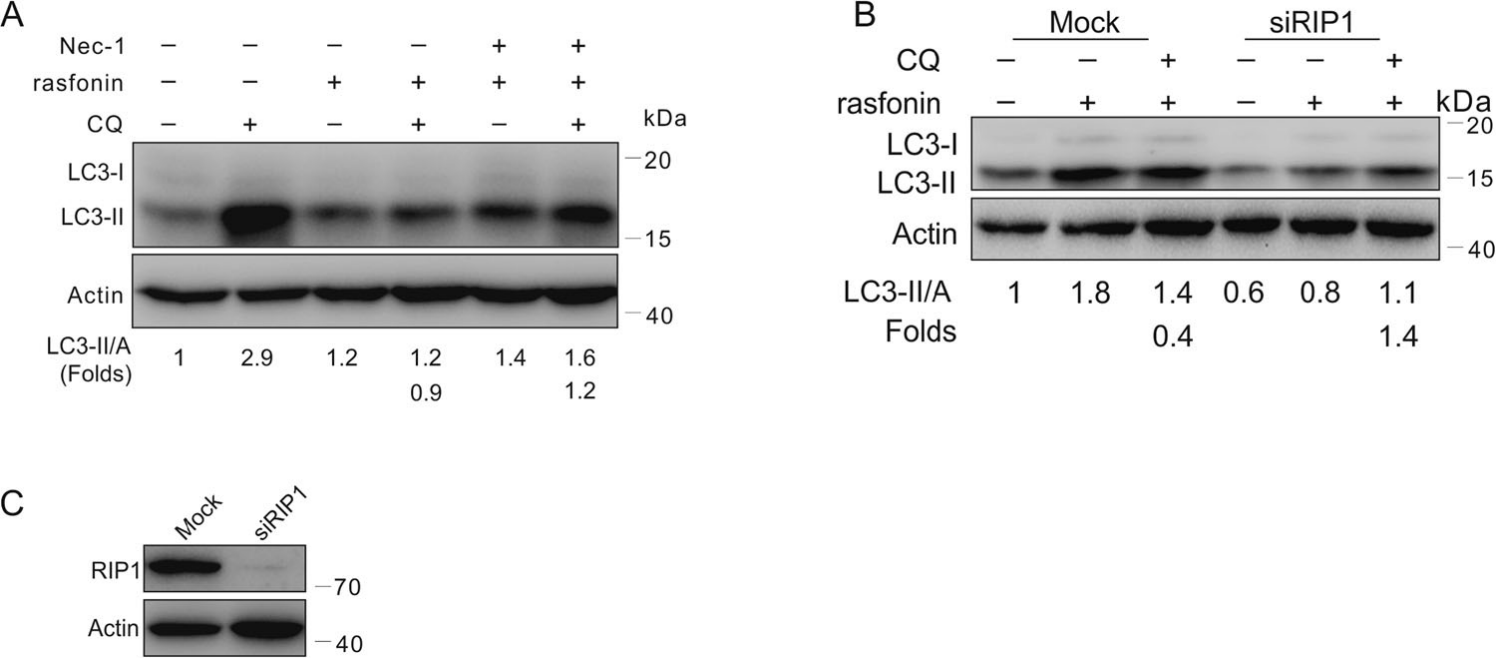
Rasfonin failed to inhibit autophagy in RIP1-deprived cells. **A** ACHN cells were treated with rasfonin (12 μM) with or without Nec-1 (30 μM) for 4 h, and then cell lysates were prepared and analyzed by immunoblotting using the indicated antibodies. **B**, **C** After transfection with the control (Mock) or RIP1 siRNA (siRIP1) for 48 h, ACHN cells were treated with rasfonin (12 μM) for 4 h. Cell lysates were analyzed by immunoblotting with the indicated antibodies. Actin was used as a loading control. Similar experiments were repeated at least three times.

Previously, we found that RIP1 was required for the activation of the autophagic process in 11'-deoxyverticillin A-treated cells [25]. In this study, we observed that Nec-1 reversed the inhibition of rasfonin on the autophagic process when the compound was used at a dose of 12 μM (Fig. 2A), suggesting that there is a switch between rasfonin-induced autophagy and necroptosis. To explore the aforementioned hypothesis, we knocked down RIP1 and detected autophagic flux in the presence of CQ. Similar to Nec-1 treatment, rasfonin failed to inhibit autophagy in RIP1-deprived cells (Fig. 2B, C), suggesting that RIP1 may play a regulatory role in switching between autophagy and necroptosis.

### Rasfonin increased the expression of EGFR, Grb2 and RIP1

**Fig. 3:**
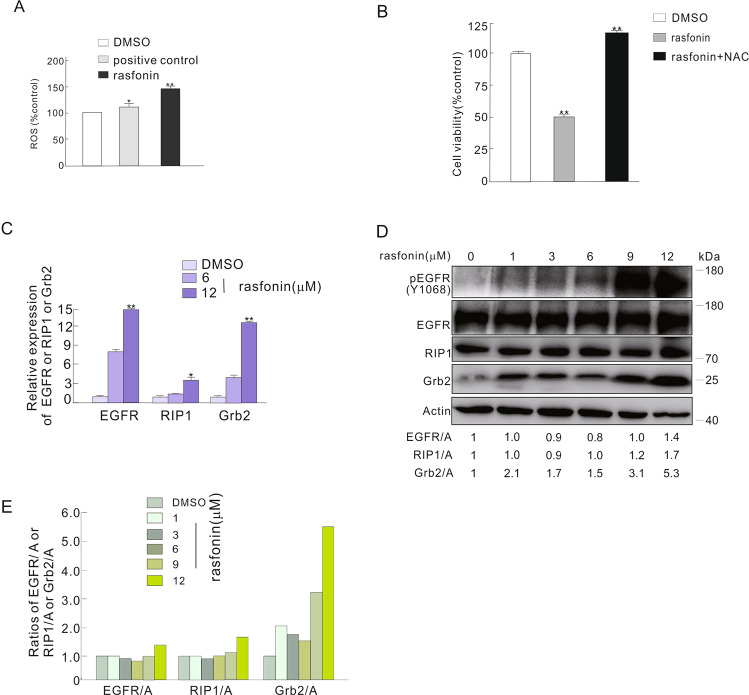
Rasfonin increased the expression of EGFR, Grb2 and RIP1. **A** The level of reactive oxygen species (ROS) was detected as described in Materials and Methods following ACHN cells were treated with rasfonin (12 μM) or positive control for 12 h. **B** ACHN cells were treated with rasfonin (12 μM) in the presence or absence of N-acetyl-L-cysteine (NAC, 0.5 mM) for 12 h; cell viability was analyzed by MTS assay as described in Materials and Methods. **C** ACHN cells were challenged with rasfonin (6 and 12 μM) for 4 h and then, the total RNA was extracted, reversed, and detected by real-time PCR. **D**, **E** ACHN cells were treated with rasfonin (0–12 μM) for 4 h, the cells were lysed and subjected to immunoblotting with the antibodies indicated. Actin was used as a loading control. Data are presented as mean ± SD and are representatives of three independent experiments. Each performed in triplicate, and the data was analyzed by *t*-test. Single asterisk denotes that the group is statistically different from the control groups (*p* < 0.05), whereas double asterisk means (*p* < 0.01). The ratios between EGFR, RIP1 or Grb2 and Actin (A) were shown below the blots (**D**). Similar experiments were repeated at least three times.

Usually, compounds derived from fungi can generate reactive oxygen species (ROS), which can induce different kinds of cell death in mammalian cells [26]. Expectedly, we observed that rasfonin challenge increased the levels of ROS (Fig. 3A), whereas N-acetyl-L-cysteine (NAC), an inhibitor of ROS [27], markedly rescued rasfonin-induced cell viability loss (Fig. 3B). A previous study revealed that ROS activate EGFR signaling [28, 29]; therefore, we assumed that rasfonin treatment might lead to the activation of the EGFR signaling pathway. To test this hypothesis, we utilized quantitative RT-PCR (qRT-PCR) to detect the expression levels of message RNA (mRNA) for EGFR and Grb2, and found that rasfonin increased the mRNA levels of for both (Fig. 3C). Interestingly, the mRNA of RIP1 was also upregulated upon the challenge with the compound (Fig. 3C). Using an immunoblotting assay, we found that high doses of rasfonin (9 and 12 μM) markedly increased the phosphorylation of EGFR, whereas it was able to upregulate protein expression of both EGFR and RIP1 at a dose of 12 μM (Fig. 3D, E). Notably, unlike EGFR and RIP1, rasfonin began to increase the level of Grb2 at a dose of 1 μM (Fig. 3D, E), whereas its treatment provided a more than 3 fold increase in Grb2 than either EGFR (3.86 fold: Grb2/actin minus EGFR/actin) or RIP1 (3.18 fold: Grb2/actin minus RIP1/actin) at the same concentration (12 μM), suggesting that, in addition to RIP1, both EGFR and Grb2 may be associated with the inhibition of rasfonin on autophagy and may play a regulatory role in rasfonin-dependent necroptosis.

### Rasfonin enhanced the interaction of Grb2 with necrosome components

**Fig. 4:**
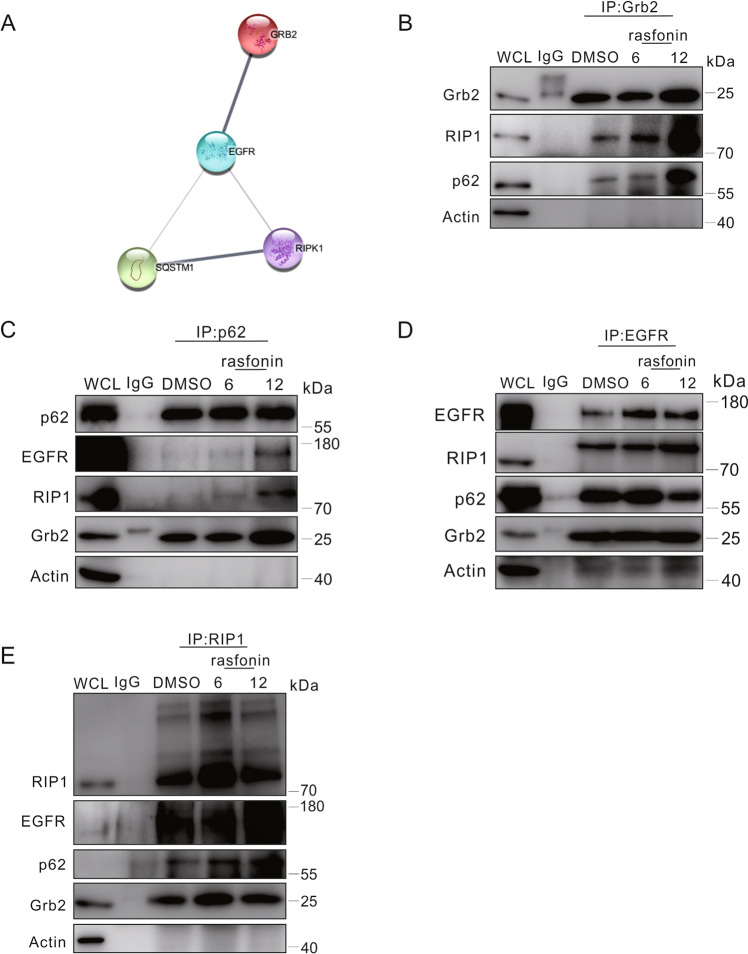
Rasfonin enhanced the interaction of Grb2 with components of necrosomes. **A** The STRING network analysis of possible interactions among EGFR, Grb2, RIP1 and p62. **B–E** ACHN cells were exposed to rasfonin (6 or 12 μM) for 6 h, and equal amounts of cell lysates were immunoprecipitated with the mouse monoclonal antibodies of Grb2, p62, EGFR, RIP1 or IgG. Immunoprecipitates were then immunoblotted for the indicated polyclonal (Grb2, p62 and EGFR) or monoclonal (RIP1) antibodies. Similar experiments were repeated twice.

Although often used as the substrate of autophagy, p62 is actually a multifunctional protein, that interacts with RIP1 and is a component of necrosome [30, 31]. As Grb2 has been proposed to be involved in zVAD.fmk and TNF-α-induced necroptosis [10], we speculated that an association between Grb2 and RIP1 or Grb2 and p62 might existed. Using the Retrieval of Interacting Genes/Proteins (STRING) web, which is a tool for exploring protein–protein interactions [32], we found potential interactions among Grb2, RIP1 and p62, whereas all these proteins including EGFR, were located in the same network (Fig. 4A). All of them are interconnected among each other and the strength of the relationship between them is indicated by the thickness of lines (Fig. 4A). To confirm the interactions among the aforementioned proteins, we next performed a co-immunoprecipitation assay. As showen in Fig. 4B, Grb2 co-immunoprecipitated with both RIP1 and p62, whereas rasfonin at the dose of 12 μM markedly enhanced their interactions. Unlike Grb2, which bound to p62 in either DMSO-control or rasfonin-treated cells, we found that p62 appeared to pull down RIP1 and EGFR only when the cells were treated with rasfonin at a dose of 12 μM (Fig. 4C), although the compound promoted more Grb2 to associate with p62 at this concentration (Fig. 4C). ROS have been considered to be involved in both necroptosis and apoptosis [33]. Given the close association between ROS and EGFR signaling [29], we assumed that rasfonin might affect the possible interaction between EGFR and components of necrosomes. Using an antibody against EGFR, we found that it pulled down both Grb2 and p62 (Fig. 4D). Nevertheless, neither of them showed an increased binding to the kinase receptor upon rasfonin challenge (Fig. 4D), whereas a relatively higher molecular band of RIP1 appeared in EGFR immunoprecipitates (Fig. 4D). In reverse immunoprecipitation using the RIP1, we observed that rasfonin enhanced the interaction between RIP1 and Grb2, whereas more EGFR appeared in its immunoprecipitate when rasfonin was used at the dose of 12 μM (Fig. 4E). Furthermore, it interacted with p62 in both DMSO- and rasfonin-treated cells (Fig. 4E). In addition to the normal molecular band, we found that RIP1 bands of higher molecular weight existed in its immunoprecipitate, suggesting that the kinase may undergo oligomerization and/or other modification under these conditions [34]. Utilizing chemical cross-linking, we observed that RIP1 might form a complex in either total cellular lysates or its immunoprecipitates (Supplement Fig. 2A, B). The above results indicated that both Grb2 and EGFR might participate in regulating rasfonin-dependent necroptosis.

### Knockdown of EGFR reversed the inhibitory effect of rasfonin on autophagy

**Fig. 5:**
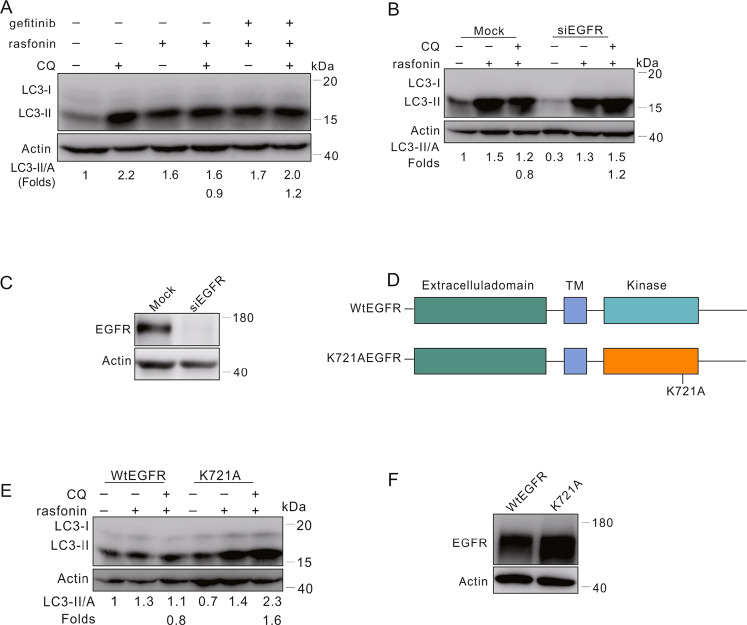
Knockdown of EGFR reversed the inhibitory effect of rasfonin on autophagy. **A** ACHN cells were treated with rasfonin (12 μM) with or without gefitinib (5 μM) for 4 h. **B** After transfection with the control (Mock) or EGFR siRNA (siEGFR) for 48 h, ACHN cells were treated with rasfonin (12 μM) for 4 h. Cell lysates in (**A**, **B**) were analyzed by immunoblotting with the indicated antibodies. **C** The efficiency of siRNA interference was determined with sample in (**B**). **D** Schematic representation of the WT EGFR and K721A EGFR. **E**, **F** HEK293T cells were transfected transiently with the WT EGFR or K721A EGFR plasmids for 36 h, and treated with rasfonin (12 μM) for 4 h. Cell lysates were analyzed by immunoblotting with the indicated antibody. Actin was used as a loading control. Similar experiments were repeated at least three times.

Autophagy plays a dual role during malignant transformation, while EGFR is able to play a key regulatory role in determining whether autophagy inhibits or promotes cancer [35]. Gefitinib is a first-generation EGFR tyrosine kinase inhibitor (TKIs) [36] and its treatment was found to enhance the autophagic process in non-small cell lung cancer. Since it increased the phosphorylation of EGFR, we speculated that rasfonin might inhibit autophagy through this kinase receptor. In the following experiment, we first used gefitinib to suppress EGFR, and found that its presence rescued autophagy in rasfonin-treated cells (Fig. 5A). To confirm the results with gefitinib, we knocked down EGFR in the cells. In contrast to the mock-control, rasfonin failed to inhibit autophagy in EGFR-deprived cells (Fig. 5B, C).

A previous study revealed that EGFR with a mutated kinase domain initiates the autophagic process upon serum starvation [37]. To further determine EGFR signaling in the regulation of rasfonin-induced autophagy, we transfected wild type (WT) EGFR and the kinase motif mutant (K721A) in HEK293T cells, which have a much lower expression of the receptor than ACHN (Supplement Fig. 2C). As expectedly, rasfonin failed to inhibit autophagy in K721A-transfected but not WT EGFR overexpressing cells (Fig. 5D-F).

### Rasfonin failed to inhibit autophagy in Grb2-depleted cells

**Fig. 6:**
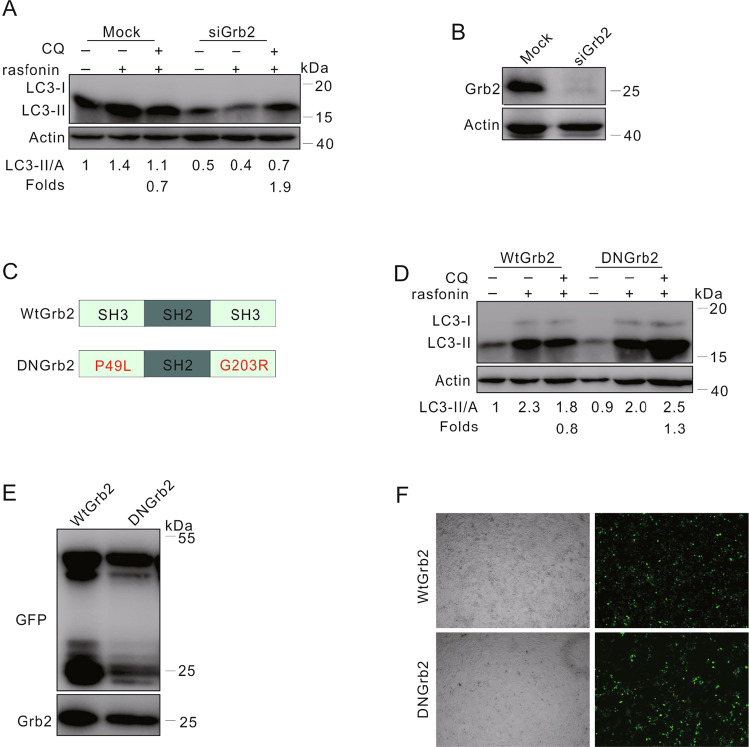
Rasfonin failed to inhibit autophagy in Grb2-depleted cells. **A** Following transfection with the control (Mock) or Grb2 siRNA (siGrb2) for 48 h, ACHN cells were treated with rasfonin (12 μM) for 4 h. Cell lysates were analyzed by immunoblotting with the indicated antibodies. **B** The efficiency of siRNA interference was determined with sample in (**A**). **C** Schematic representation of the WT Grb2 and mutant DN Grb2. **D**, **E** HEK293T cells were transfected transiently with the WT Grb2 or DN Grb2 plasmids for 36 h, and treated with rasfonin (12 μM) for 4 h. Cell lysates were analyzed by immunoblotting with the indicated antibody. Actin was used as a loading control. **F** The transfection efficiency was detected and acquired by using fluorescence microscope. Similar experiments were repeated at least three times.

In human umbilical vascular endothelial cells (HUVECs), knockdown of Grb2 was found to inhibit either pterostilbene or upregulation of the active form of Rab13-induced autophagy [38]. More recently, a study demonstrated that microRNA-378 (miR-378) attenuated autophagy and inhibited AKT/mTOR signaling downregulating Grb2 [39]. Since Grb2 is a downstream adapter of EGFR, we wondered whether the expression of Grb2 could affect rasfonin-dependent autophagy. Similar to EGFR depletion, rasfonin was unable to inhibit the autophagic process in Grb2-depleted cells (Fig. 6A, B). To confirm the aforementioned results, we transfected HEK293T cells with either WT or SH3 domain mutated Grb2 (DN Grb2, a function-loss mutant), and found that overexpression of WT Grb2 but not DN Grb2 suppressed the rasfonin-depended autophagic process (Fig. 6C-F).

### Grb2 mediated the expression of RIP1 and was involved in rasfonin-induced necroptosis

**Fig. 7:**
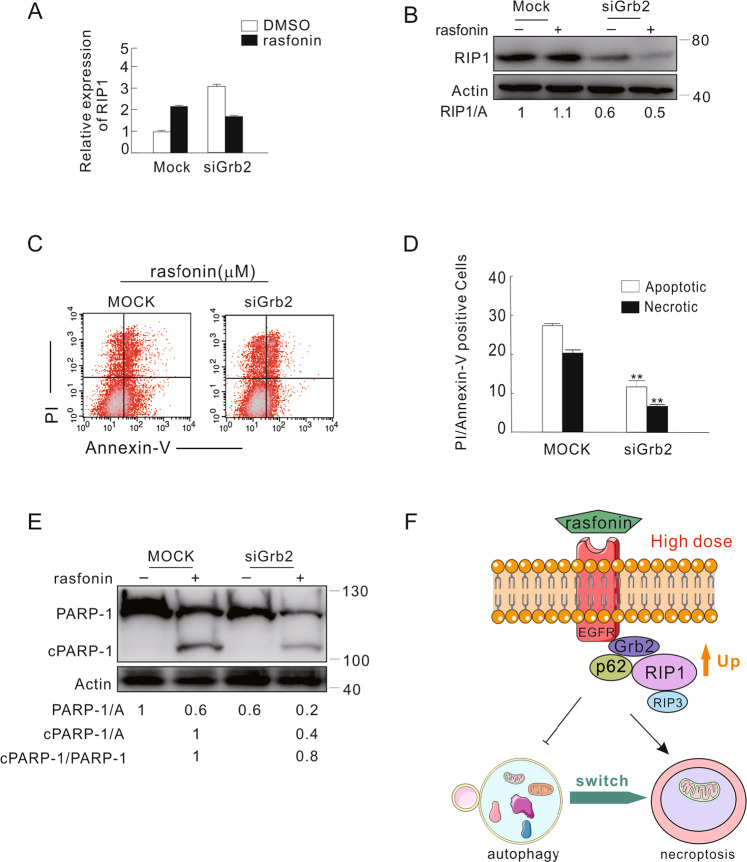
Grb2 mediated the expression of RIP1 and was involved in rasfonin-induced necroptosis. **A–E** After transfection with the control (Mock) or Grb2 siRNA (siGrb2) for 48 h, ACHN cells were exposed to 12 μM rasfonin for 6 h, and the total RNA was extracted, reversed, and detected by real-time PCR (**A**); cell lysates were analyzed by immunoblotting with the indicated antibodies, Actin was used as a loading control (**B**); the apoptosis and necrosis induced by rasfonin were determined by flow cytometry (**C** and **D**). Apoptotic: AV-positive and PI-negative; necrotic: PI-positive. The data are presented as mean ± S.D. from three independent experiments. The double asterisk denotes the group is statistically different from the control groups (*P* < 0.01). **E** Cell lysates in (**B**) were analyzed by immunoblotting with the indicated antibodies, and ratios between cleaved PARP-1 (cPARP-1) and PARP-1, or between cPARP-1 and Actin (A), or between PARP-1 and Actin (A) were shown below the blots. Similar experiments were repeated at least three times. **F** Schematic mechanism of EGFR/Grb2 in rasfonin-dependent autophagy and necroptosis.

Although it has not been directly proven, Grb2 is proposed to be involved in necroptosis [10]. Given the RIP1 interacts with Grb2 and EGFR, we next examined whether Grb2 was able to regulate the expression of RIP1 in the cells with or without rasfonin treatment. Indeed, we found that the expression of RIP1 mRNA was upregulated in Grb2-silenced cells, whereas rasfonin treatment markedly increased the mRNA expression of RIP1 in mock-control cells but not in Grb2-depleted cells (Fig. 7A). Utilizing immunoblotting, however, we found that knockdown of Grb2 failed to increase the protein levels of RIP1 in both DMSO-control and rasfonin-treated cells, suggesting that the loss of Grb2 may affect the translation of RIP1. In contrast, rasfonin upregulated the protein expression of RIP1 in mock-control cells (Fig. 7B). To determine whether Grb2 was involved in regulating rasfonin-activated necroptosis, we performed flow cytometry assays following Grb2 silencing. As shown in Fig. 7C, rasfonin-induced necrosis was markedly decreased in Grb2-deprived cells (Fig. 7C, D). Unexpectedly, the loss of Grb2 also reduced the induced apoptosis and the cleavage of poly (ADP-ribose) polymerase-1 (PARP-1) (Fig. 7E), which is often used as an indicator of caspase-dependent apoptosis [5]. Given the Nec-1 is able to inhibit rasfonin-promoted necrosis, we assumed that Grb2 was likely involved in rasfonin depended necroptosis.

## Discussion

In the present study, we directly proved that either EGFR or Grb2 was able to interact with p62 and RIP1, both of which were demonstrated to participate in the regulation in necroptosis, whereas the reduction of rasfonin-induced necrosis in Grb2-depleted cells confirmed that Grb2 did play a regulatory role in the programmed necrotic process.

Compounds from fungi often produce ROS, which have a signaling role in a variety of biological systems and induce programmed cell death or necrosis [26]. While the regulatory role of ROS in other modes of cell death has been widely investigated [40], its impact on necrotic signaling and necroptosis is less clear. Recently, a study revealed that ROS activated RIP1 autophosphorylation on serine residue 161 to ensure the effective induction of necroptosis [41]. It is well known that ROS can transactivate EGFR to cause activation of the Ras/mitogen-activated protein kinase (MAPK) pathway [28], whereas Ras can also be the upstream signal for AKT, which is involved in regulating necroptosis [34, 42]. Thus, we reasonably believe that rasfonin induced necroptosis by generating ROS, consequently leading to the activation of EGFR/Ras signaling, in which Grb2 is well known to play a critical role. It should be noted that, unlike Grb2, the EGFR antibody failed to pull down RIP1 at its normal molecular weight. Therefore, we assumed that the interaction between EGFR and Grb2 could convey necrotic signaling to RIP1 to induce necroptosis upon rasfonin stimulation. Since Grb2 also binds to either TNFR1 or FasL [10, 43], whether these two proteins are involved in rasfonin-induced necroptosis awaits further investigation. Although knockdown of Grb2 increased the mRNA expression of RIP1, we failed to observe an increase in its protein level, suggesting that loss of the adapter protein may affect translation of the latter. Consequently, the reduced expression of RIP1 should have disrupted the formation of nucleosomes in response to rasfonin challenge.

In addition to regulating necroptosis, RIP1 is also involved in the mediation of autophagy [44]. Our previous study revealed that RIP1 and PARP-1 are required for the activation of the autophagic process upon challenge with 11'-deoxyverticillin A, another compound isolated from fungi [25]. RIP1 was found to participate in regulating both basal and induced autophagic processes. Moreover, in L929 fibroblast cells, the role of RIP1 in autophagic cell death has been demonstrated [45]. Given that RIP1 was able to mediate either autophagy or necroptosis induced by rasfonin, its role in programmed cell death was possibly decided or closely associated with the strength of any stimulus. Additionally, RIP1 was certainly found to play a regulatory role other than necroptosis; indeed, it has been found to maintain epithelial homeostasis by inhibiting both apoptosis and necroptosis [46]. Therefore, RIP1 may perform multiple functions in regulating cellular physiology and physiopathology. Concerning its role in necroptosis, we reasonably believe that the role of RIP1 could be context dependent.

Although often utilized as a substrate of autophagy, p62 is a protein that possesses many biological functions and acts as a binding partner of various proteins [14]. Importantly, it plays a critical role in selective autophagy and connects two degradation pathways (autophagy/lysosome and proteasome/ubiquitin) in eukaryotic cells [47]. In both cancer cells and neurons, p62 can serve as a scaffold to modulate the mode of programmed cell death [48]. Upon stimulation of necroptosis, p62 forms a complex with RIP1-RIP3, promotes the binding of RIP1 and RIP3, and induces subsequent MLKL phosphorylation, resulting in programmed necrosis [15]. In the present study, we found that, unlike binding to Grb2, p62 only pulled down RIP1 and EGFR when rasfonin was used at a relatively high dose. Thus, it might cooperate with Grb2 to recruit more EGFR and/or RIP1 for the activation of necroptosis. Since they interact with either p62 or RIP1, the direct involvement of EGFR and Grb2 in the formation of nucleosomes requires further investigation.

In conclusion, our study identified that both EGFR and Grb2 interacted with the components of necrosomes to participate in rasfonin-induced necroptosis (Fig. 7F). The data presented here broaden our knowledge about programmed necrosis and enable us to better understand the regulatory mechanism of necroptosis and autophagy.

## Materials and methods

### Chemicals and antibodies

Chloroquine diphosphate salt (CQ, C6628), gefitinib (Gef, SML1657), necrostatin-1 (Nec-1, 480065), N-acetyl-L-cysteine (NAC, 1009005) and polyclonal antibodies against LC3 (L7543) were purchased form Sigma-Aldrich (St. Louis, MO, USA). Cell Signaling Technology (Boston, MA, USA) provided antibodies against p-EGFR (Tyr1068, 3777), EGFR (2232) and RIP1 (3493). Purified Mouse anti-RIP (610458) was acquired from BD Transduction Laboratories (Franklin, NJ, USA). Anti-Grb2 antibodies (ab49876 and ab32037) was obtained from Abcam (Cambridge, MA, UK) and monoclonal antibody of Grb2 (sc-8034) was purchased from Santa Cruz Biotechnology. Proteintech (Wuhan, China) supplied antibody against SQSTM/p62(18420-1-AP) and EGFR (18986-1-AP). ZhongShanJinQiao Biocompany (Beijing, China) produced anti-actin (TA-09). Alexa Fluor® 488 Goat anti-Mouse IgG (H + L) secondary antibody (A-11001) were acquired from Molecular Probes (Eugene, OR, USA). MTS reagent powder (G1111) was obtained from Promega Corporation (Madision, WI, USA). ROS kit (S0033) was purchased for Beyotime (Shanghai, China). Small interference RNA (siRNA) of RIP1 (sc-36426 and L-004445-00), EGFR (sc-29301 and L-003114-00) and Grb2 (sc-29334 and L-019220-00) were obtained from Santa Cruz Biotechnology and Dharmacon along with control siRNA.

### Cell culture and immunoblotting analysis

The experiments were performed as previously decribed [26, 49]. ACHN cells were purchased from the American Type Culture Collection (ATCC, Manassas, VA, USA). HEK293T cells were kindly provided by Dr. Alexander Sorkin (Faculty, Cell Biology, University of Pittsburgh, 4200 Fifth Avenue Pittsburgh, PA 15260). ACHN and HEK293T cells were cultured in DMEM medium (HyClone; SH20022.01B) containing 1% antibiotics and 10% fetal bovine serum (GIBCO, Grand Island, NY, USA). Transient transfection was performed using Lipofectamine 2000 (Invitrogen) or Attractene (QIAGEN) according to manufacturer’s protocol when cells were grown to 80% conflency. After 36 h transfection, the cells were spilt, cultured overnight and exposed to the indicated stimulations. For siRNA interference, cells were cultured to 30% conflency in the media without antibiotics were transfected using DharmaFECT (Dharmacon, T2001) according to manufacturer’s instructions. After transfection for 48 h, the cells were spilt, grew overnight and then exposed to the indicated stimulations. Whole cell lysates were prepared with lysis using Triton X-100/glycerol buffer (4 mM EDTA, 2 mM EGTA, 1 mM dithiothreitol, 50 mM Tris-HCl, pH 7.4, 1% Triton X-100, 1% SDS, and protease inhibitors), then separated on an SDS-PAGE gel and transferred onto a PVDF membrane. Immunoblot analyses were performed using the appropriate primary antibodies and horseradish peroxidase-conjugated suitable secondary antibodies. The proteins were visualized by chemiluminescence (Pierce Chemical).

### Immunoprecipitation

The cell extract was prepared with lysis using Triton X-100/glycerol buffer as described above. EGFR, p62, RIP1 and Grb2 were immunoprecipitated over 3 h in 4 °C and then incubated for 1 h in Protein A-Sepharose or Protein G-Sepharose (Vigorous Biotechnology, Beijing, China). Cell lysates were electrophoresed on SDS-PAGE and subjected to immunoblotting analysis. The method was performed as previous [50].

### Chemical cross-linking

Chemical cross-linking was performed as previously described [5]. Briefly, the cells were seeded in 100 mm-dishes with 70–80% confluence, and then, the culture was carried out for 12 h before cells collection and washing following the indicated treatment. After resuspending the pellet with PBS to 5 × 10^6^ cells/ml, 4% paraformaldehyde was added to the final concentration of 0.5%, and then cells were incubated for 30 min at 37 °C. Following addition of 2.5 M glycine (pH 3.0) to the final concentration of 125 mM and incubation at room temperature (RT) for 5 min, cells were centrifuged at 2,000 rpm for 5 min. After washing twice with PBS, Triton X-100/glycerol buffer was added to acquire the whole cell lysates, which were then used in the following immunoprecipitation experiments. For the samples acquired after chemical cross-linking, the denaturation temperature was set at 70 °C instead of 96 °C, to avoid depolymerization of the complexes.

### Cell viability assay (MTS)

Cell viability assay was performed as previously described [50]. Briefly, the cells were grown in 96 well plates (7,500 cells per well) in 100 µL complete culture media. After overnight culture, the cells were replaced with complete medium of phenol red free, which was added either by drug-free or with rasfonin or other chemicals (Nec-1 and NAC). The cells were incubated for indicated period and the cell viability was detected by CellTiter 96 Aqueous Non-Radioactive Cell Proliferation Assay (Promega).

### ROS assay

ROS assay was performed as previously described [26]. ACHN cells were divided into 96 well plates and incubated overnight at 100 μL per well. DCFH-DA was diluted with a serum-free medium at 1:1000 to a final concentration of 10 μmol/l. Remove the cell culture medium and add an appropriate amount of diluted DCFH-DA. This is recommended to add the volume to cover the cell. Incubate for 20 min in a 37 °C cell incubator. The cells were washed three times in a serum-free cell culture medium and completely remove DCFH-DA which did not enter the cells. Microplate reader (488 nm excitation wavelength, 525 nm emission wavelength), each sample set up three holes, the same test repeated three times.

### Fluorescence microscopy

The experiment was done as previously decribed [49]. Either HEK293T or ACHN cells were split and cultured on coverslips for 12 h before addition of rasfonin for the time indicated. Freshly prepared 4% paraformaldehyde was utilized to fix the cells at room temperature (RT) for 15 min. After washing three times with PBS, the cells were blocked with PBS containing 3% BSA for 1 h, and then incubated with the indicated antibodies, stained with Alex Fluor 488 secondary antibodies and visualized using Fluorescence microscopy (Zeiss).

### Electron microscopy

Electron microscopy was performed as described [5]. Shortly, the sample was washed three times with PBS, trypsinized, and collected by centrifuging. The cell pellet was immobilized with 4% paraformaldehyde at 4 °C overnight, post-fixed with 1% OsO_4_ in cacodylate buffer at RT for 1 h, and dehydrated stepwise with ethanol. The dehydrated pellets were rinsed with propylene oxide at RT for 30 min and embedded in Spurr resin for sectioning. Images of thin sections were observed under a transmission electron microscope (JEM1230, Japan).

### Flow-cytometry assay

The ACHN cells were challenged by rasfonin, then trypsinized and harvested (keeping all floating cells), washed with cold PBS buffer. After washing, the cells were incubated with fluorescein isothiocyanate-labeled annexin V (FITC) and propidium iodide (PI) as instructed in the Annexin-V-FITC Apoptosis Detection Kit (Biovision Inc., Milpitas, CA, USA, K101-100) and analyzed by flow cytometry (FACSAria, Becton Dickinson, Franklin Lakes, NJ, USA). The cells with annnexin V-positive and PI-negative staining were calculated as apoptotic, whereas PI-positive staining was considered as necrotic. The method was performed as previous [17, 18].

### Reverse transcription and real-time PCR

The total cellular RNA was extracted using TRIzol reagent (Invitrogen; 15596-018) according to the manufacturer’s protocol, and the RNA integrity was confirmed by electrophoresis of 1% agarose gel stained ethyl bromide. 1 μg of the total RNA was reversely transcribed using PrimeScriptTM RT reagent Kit (TaKaRa; DRR037A). Real-time PCR was carried out using a SYBR real-time PCR kit (Sigma-Aldrich) in an Eppendorf Mastercycle EP (Eppendorf AG 22331; Hamburg). Primer sequences used for amplification were as follows: RIP1 upstream primer, 5'- GGA GAT TGG TGG GAC GAG-3'; downstream primer, 5'- GTG AAG CCC AGT TTA CGG -3'; EGFR upstream primer, 5'- TGT GCC CAC TAC ATT GAC G -3'; downstream primer, 5'- GGG ATC TTA GGC CCA TTC -3'; Grb2 upstream primer, 5'- AAA AGA CGG CTT CAT TCC -3'; downstream primer, 5'- GTG CTG CAC ATC GTT TCC -3'; *β*-actin upstream primer, 5'-GCC TGA CGG CCA GGT CAT CAC-3'; downstream primer, 5'-CGG ATG TCC ACG TCA CAC TTC-3'. Expression of *β*-actin was used as the internal control. The method was performed as previous [5].

### Statistical analysis

The linear range of chemiluminescence signals of immunoblotting images were analyzed and validated in order to perform quantifications were carried out through utilizing densitometry. Statistical significance was analyzed using one-way ANOVA and the Student-Newman-Keuls post-hoc test. Results of multi-plates enzyme immunoassay was shown as mean ± SD [49].

## Supplementary information

Supplemental Information

Original full length western blots

## Data Availability

All data generated or analyzed during this study are included in this published article and its supplementary information files.
