# Supplementary material for: Grb2 interacts with necrosome components and is involved in rasfonin-induced necroptosis
Source: Cell Death Discov. 2022 Jul 13;8:319. doi: 10.1038/s41420-022-01106-1 (PMC9279413; doi:10.1038/s41420-022-01106-1)
Supplement: Supplementary file 1 — Supplemental Information [file 41420_2022_1106_MOESM1_ESM.doc]

**Grb2 interacts with necrosome components and is involved in rasfonin-induced necroptosis**

Bolin Hou,^1,2^ Haiwen Huang,^3^ Yueqian Li, ^4,5^ Jingnan Liang, ^5^ Zhijun Xi,^3^ Xuejun Jiang, ^1^ Ling Liu ^1^, * and Erwei Li^1, 5^*

^1^State Key Laboratory of Mycology, Institute of Microbiology, Chinese Academy of Sciences, Beijing 100101, China

^2^CAS Key Laboratory of Microbial Physiological and Metabolic Engineering, Institute of Microbiology, Chinese Academy of Sciences, Beijing 100101, China

^3^Department of Urology, Peking University First Hospital, Beijing 100034, China

^4^School of Pharmacy and State Key Laboratory of Applied Organic Chemistry, Lanzhou University, Lanzhou 730000, China

^5^Institutional Center for Shared Technologies and Facilities, Institute of Microbiology, Chinese Academy of Sciences, Beijing 100101, China

*Correspondence to: Erwei Li; Email: [liew@im.ac.cn](mailto:liew@im.ac.cn) and Ling Liu; Email: [liul@im.ac.cn](mailto:liul@im.ac.cn)

**Running title:** The involvement of EGFR/Grb2 in rasfonin-dependent autophagy and necroptosis

**This Supplementary information includes:**

**Supplementary Figure: Figure 1-2**

**Supplemental Figure 1**


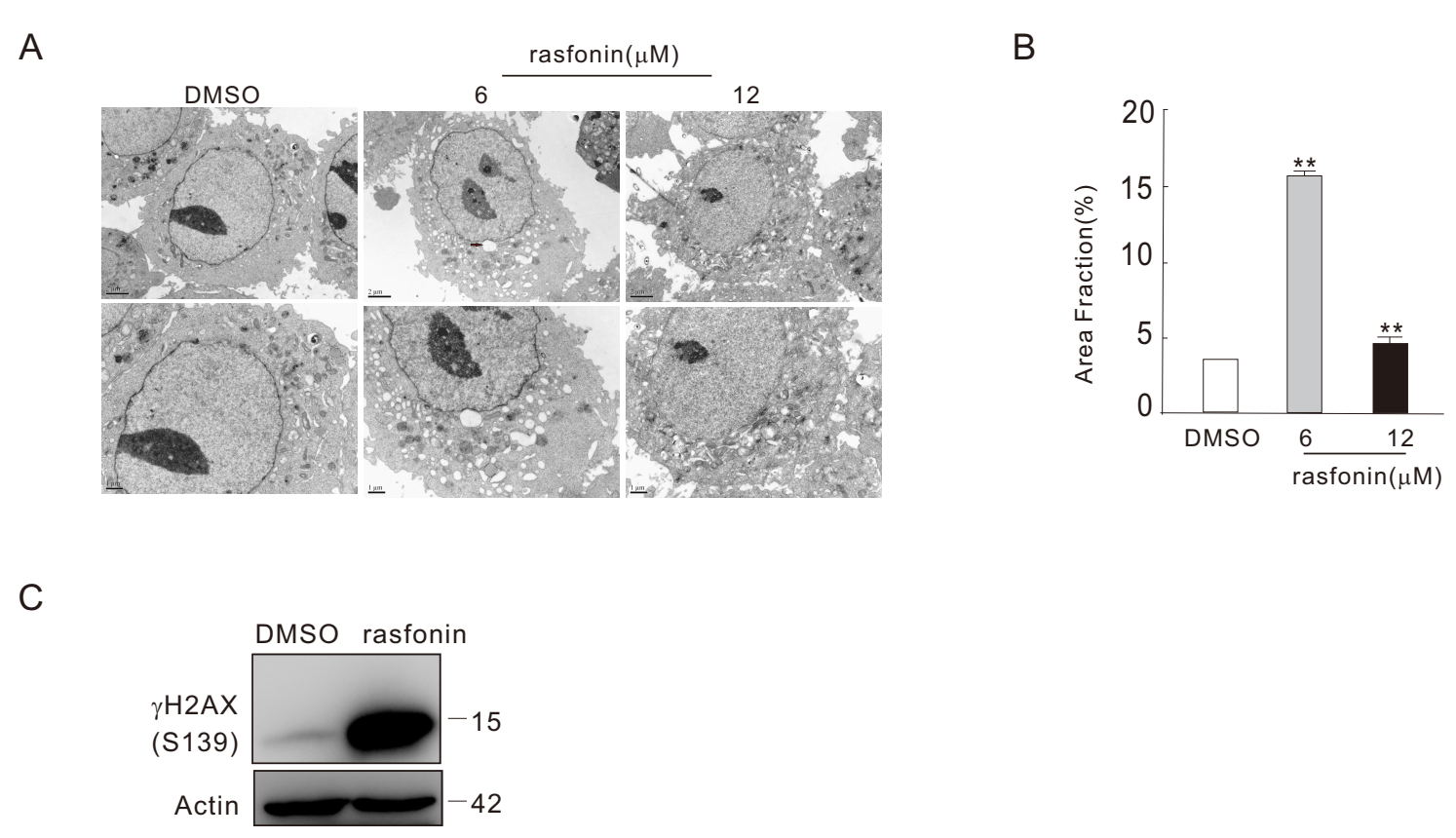


**Supplemental Figure 1. High concentration of rasfonin inhibited autophagy and upregulated γH2AX expression.** (**A** and **B**) Transmission electron microscopy was performed in ACHN cells following treatment of rasfonin (6 or 12 μM) for 6 h. The morphometric analysis of the area fraction between autophagosomes and cytoplasm was calculated by using the Photoshop software. The data of the area ratio were non-normally distributed, and are presented as the mean of at least 20 cells counted for each group. Arrow: membrane vacuoles. (**C**) ACHN cells were treated with rasfonin (12 μM) for 6 h, the cells were lysed and subjected to immunoblotting with the antibodies indicated. Actin was used as a loading control. Similar experiments were repeated at least three times.

**Supplemental Figure 2**


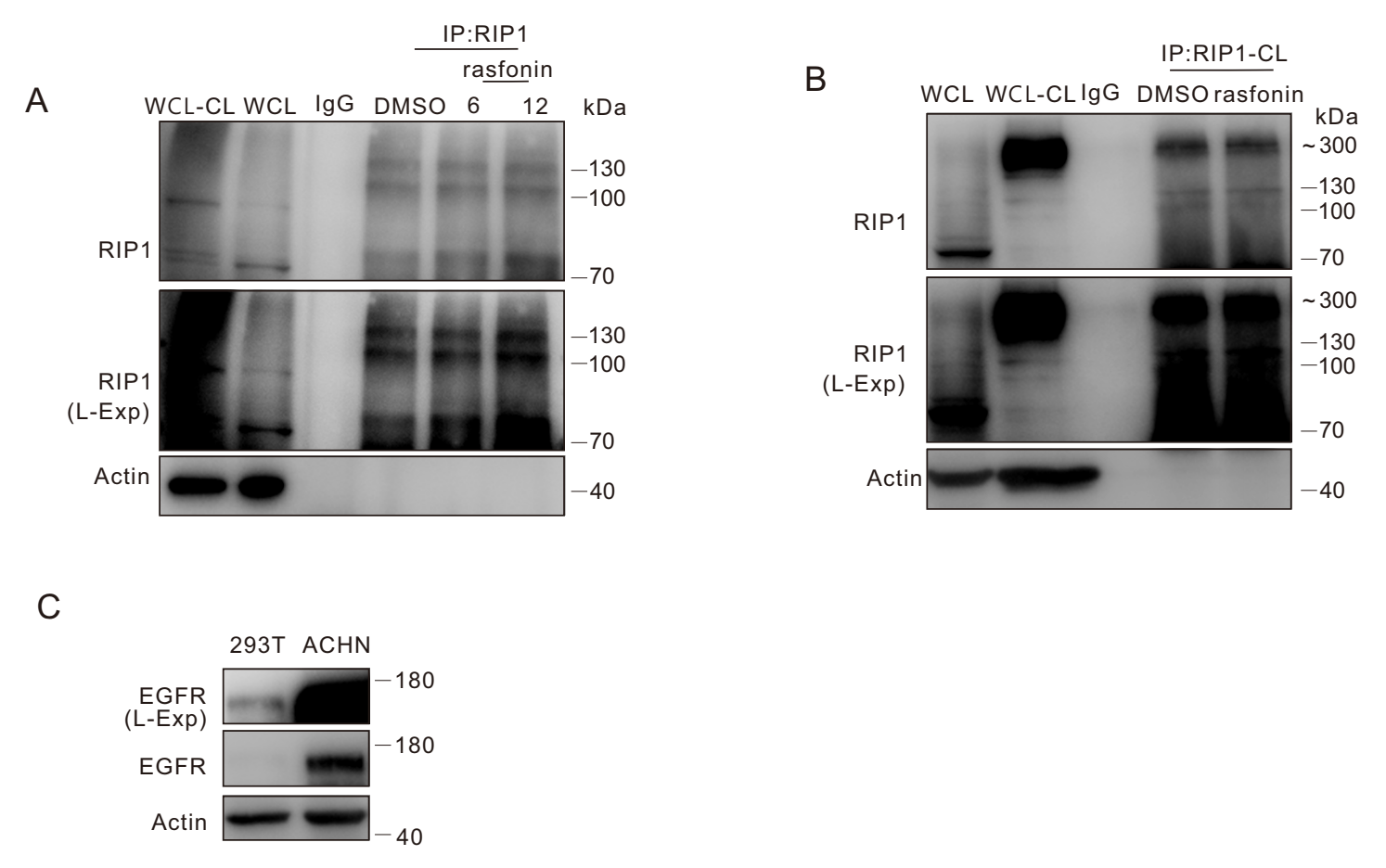


**Supplemental Figure 2. RIP1 formed higher molecular bands.** (**A**) ACHN cells were exposed to rasfonin (6 or 12 μM) for 6 h, and equal amounts of cell lysates were immunoprecipitated with the antibody of RIP1 or IgG. Immunoprecipitates were then immunoblotted for the antibodies indicated. (**B**) Following treatment with rasfonin (12 μM) for 6 h, ACHN cells were subjected to chemical cross-linking and immunoprecipitation with the RIP1 antibody. The whole cell lysates (WCL) and immunoprecipitates were analyzed by immunoblotting with the antibodies indicated. Similar experiments were repeated twice. CL: Cross linking. (**C**) The lysates of ACHN and HEK293T cells were analyzed by immunoblotting with antibodies indicated. Actin was used as a loading control. Similar experiments were repeated at least three times.
