# Supplementary material for: Grb2 interacts with necrosome components and is involved in rasfonin-induced necroptosis
Source: Cell Death Discov. 2022 Jul 13;8:319. doi: 10.1038/s41420-022-01106-1 (PMC9279413; doi:10.1038/s41420-022-01106-1)
Supplement: Supplementary file 2 — Original full length western blots [file 41420_2022_1106_MOESM2_ESM.docx]

**Original full length western blots**

**PVDF membranes were cut into stripes for different antibodies staining. All images were exposed by Thermo fisher Luminometer.**

Figure 1

A


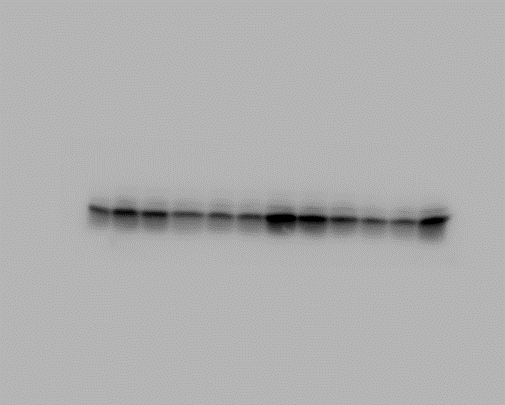

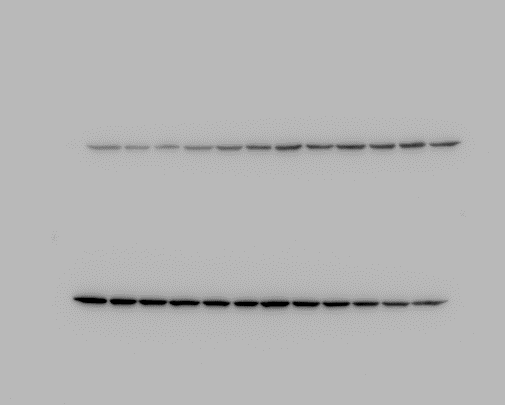


LC3 Actin

Figure 2

A


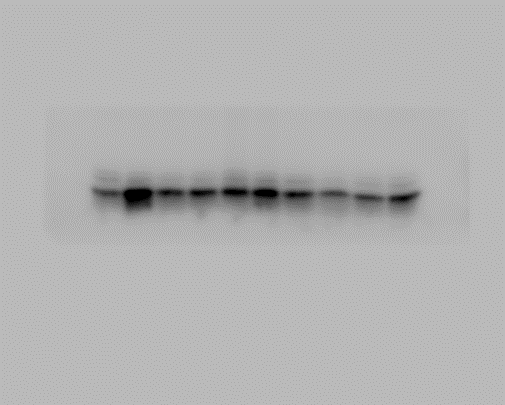

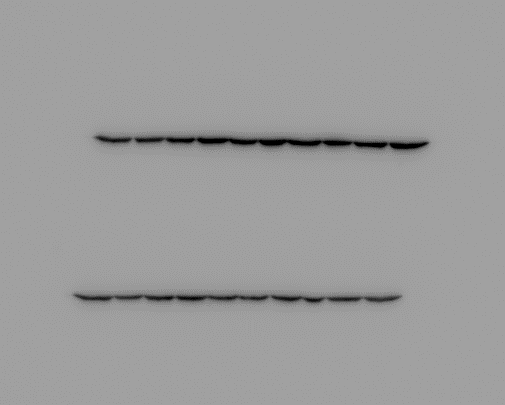


LC3 Actin

B


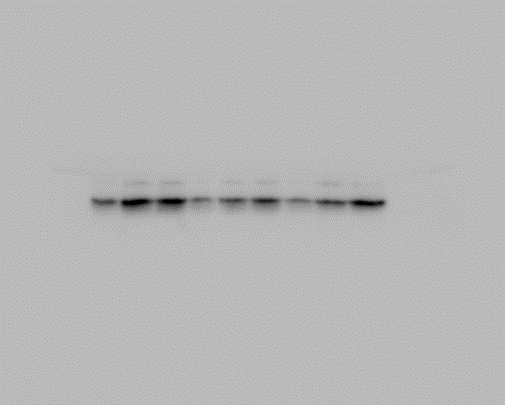

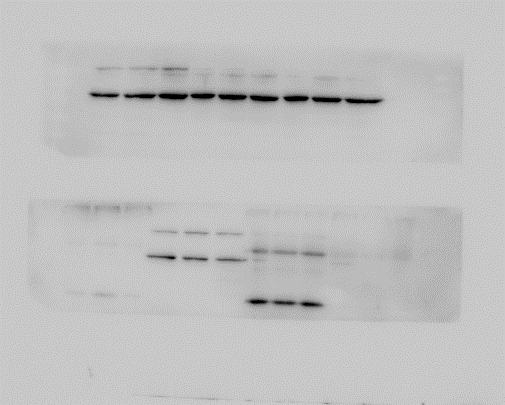


LC3 Actin

C


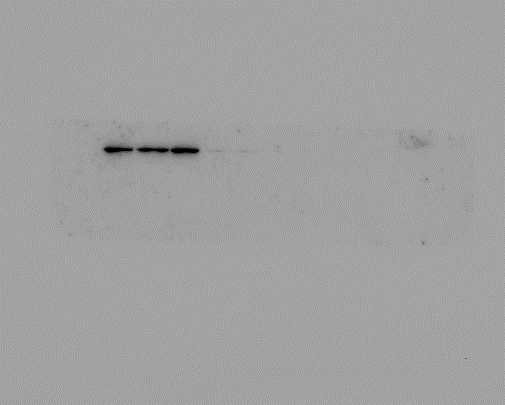

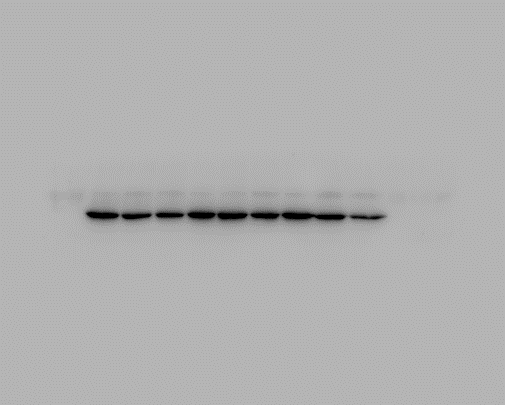


RIP1 Actin

Figure 3

D


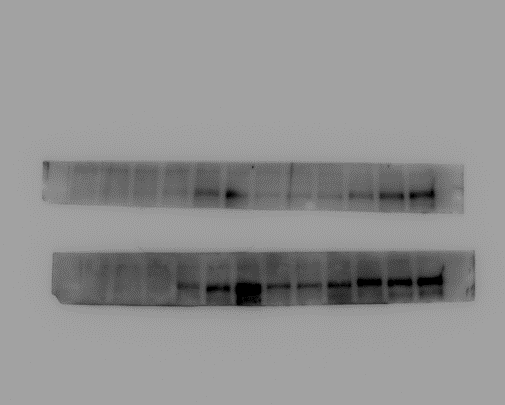

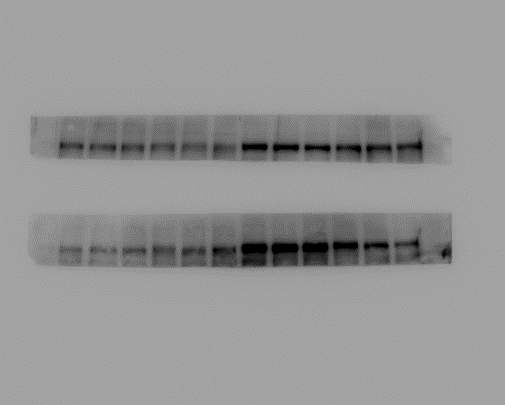


pEGFR(Y1068) EGFR


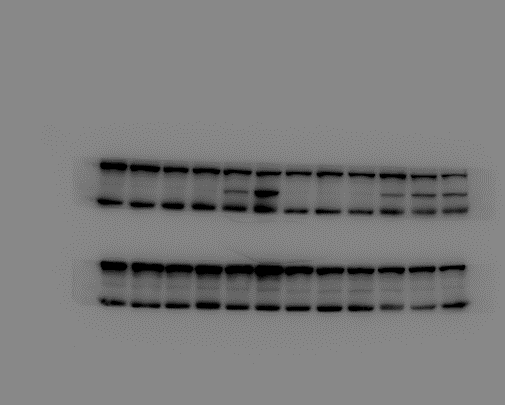

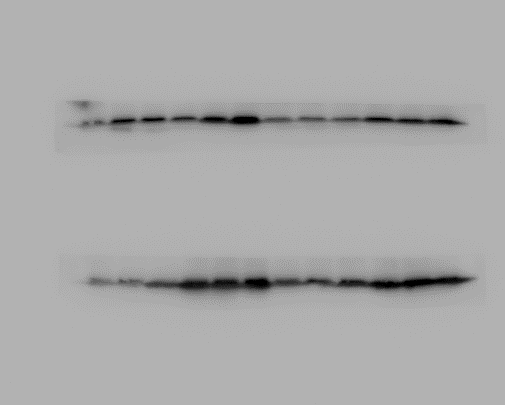


RIP1 Grb2


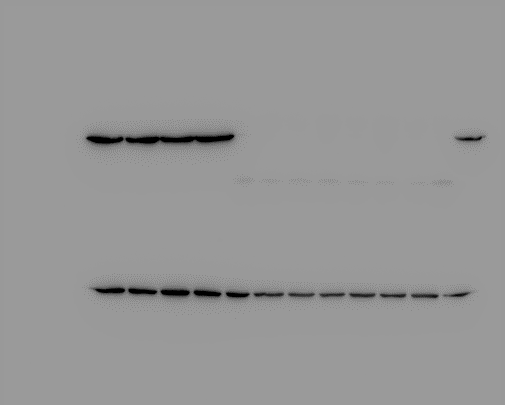


Actin

Figure 4

B


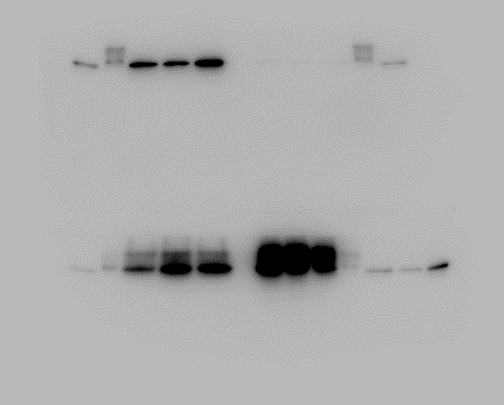

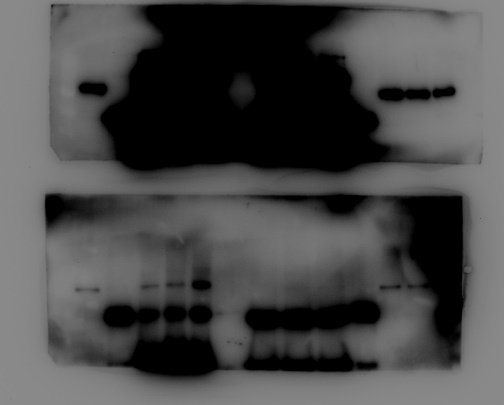


Grb2 RIP1


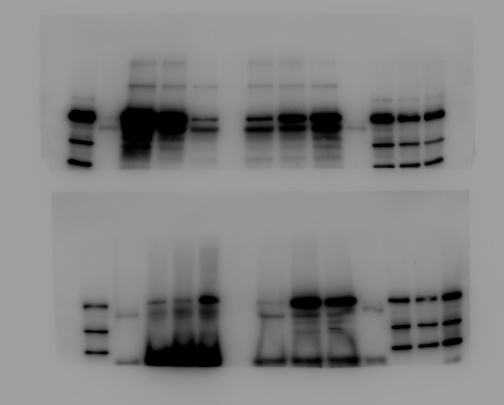

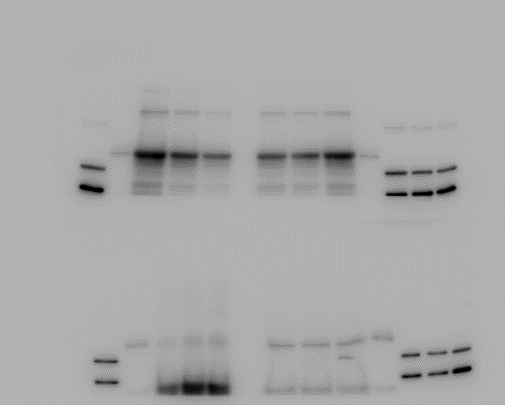


p62 Actin

C


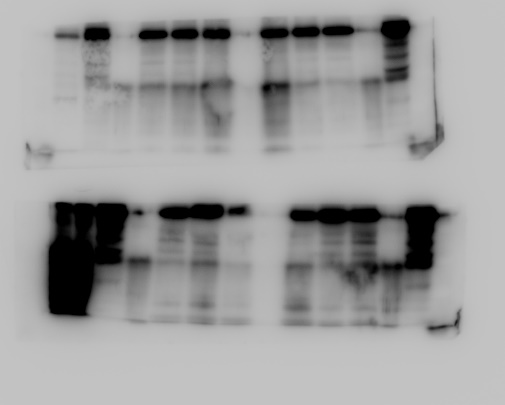

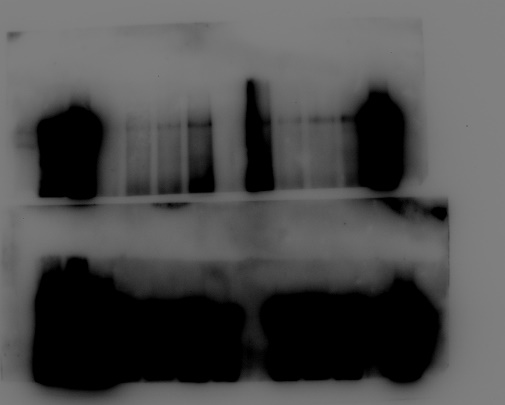


p62 EGFR


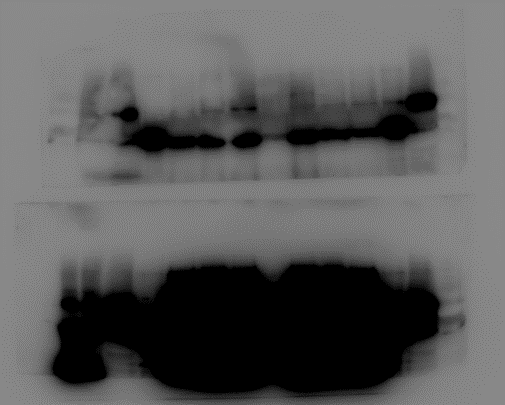

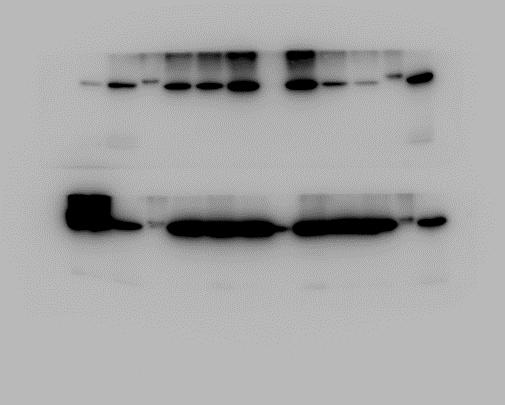


RIP1 Grb2


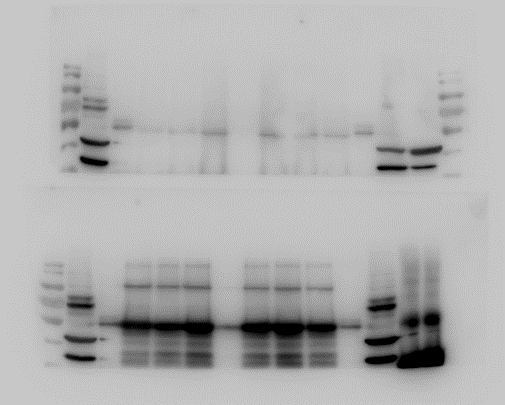


Actin

D


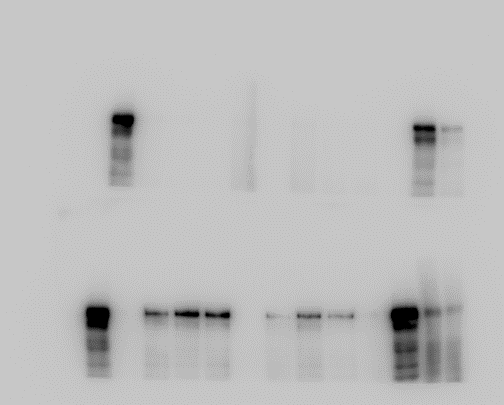

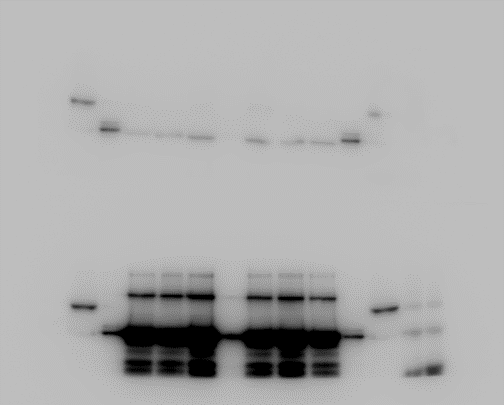


EGFR RIP1


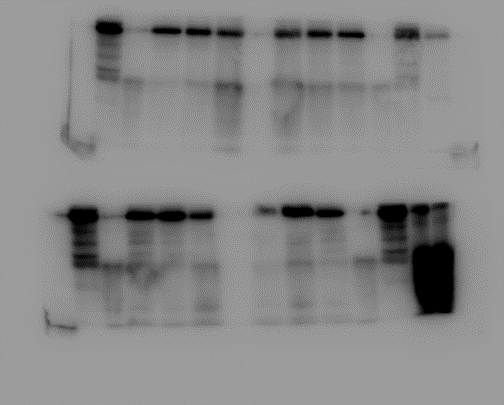

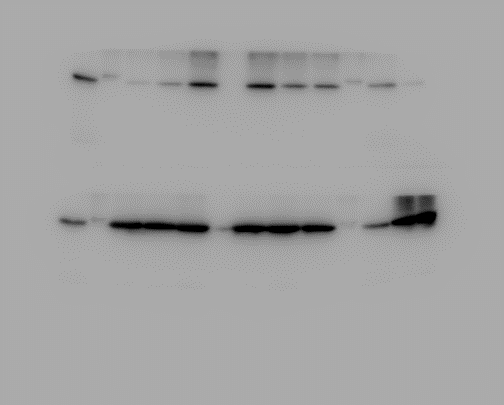


p62 Grb2


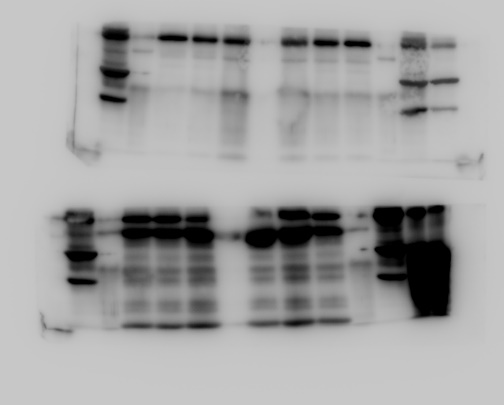


Actin

E


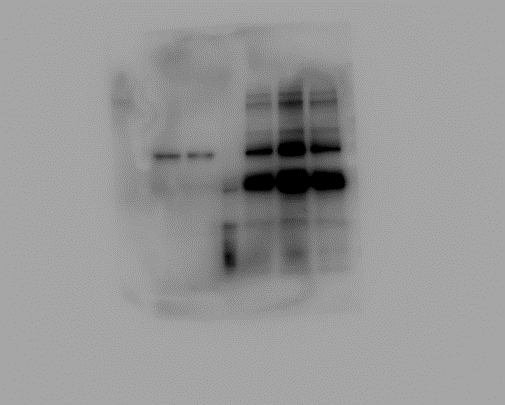

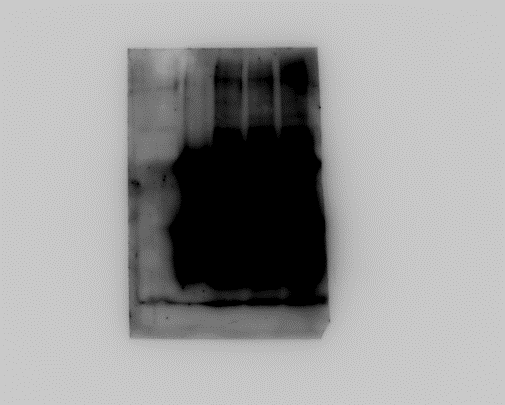


RIP1 EGFR


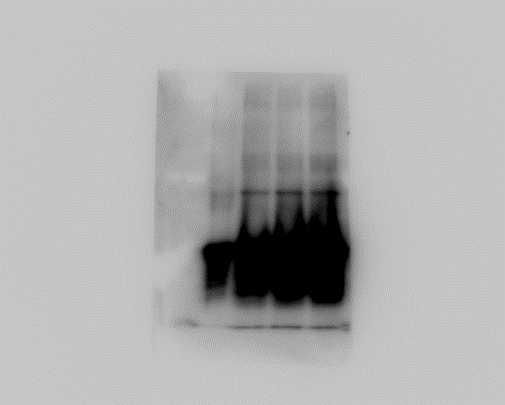

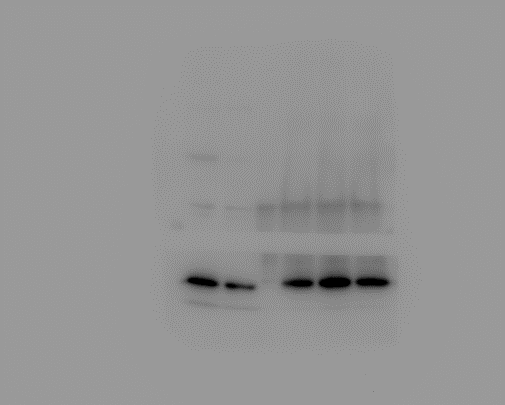


p62 Grb2


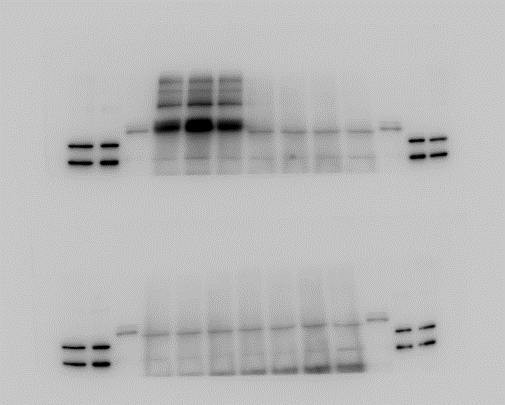


Actin

Figure 5

A


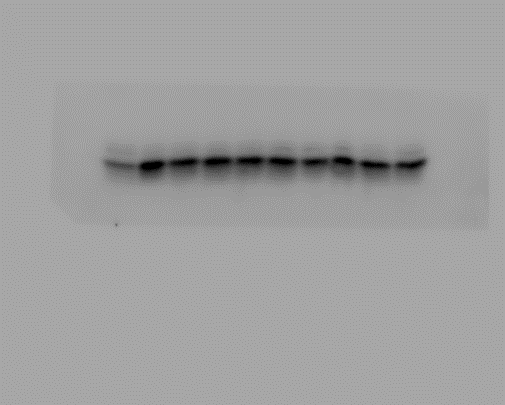

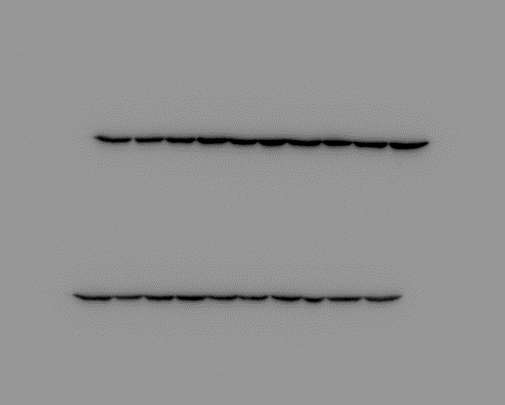


LC3 Actin

B


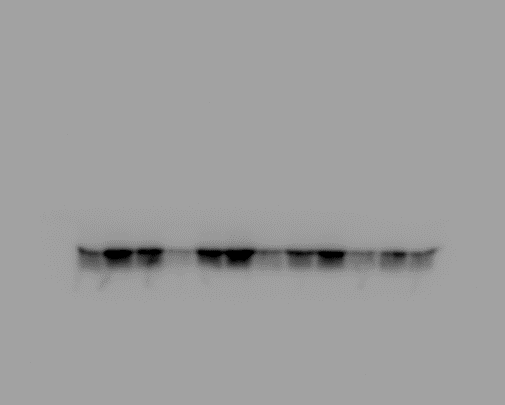

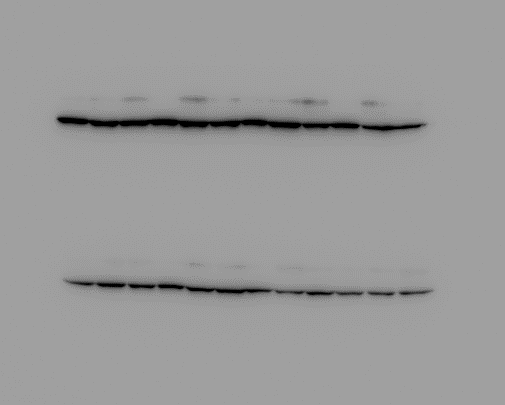


LC3 Actin

C


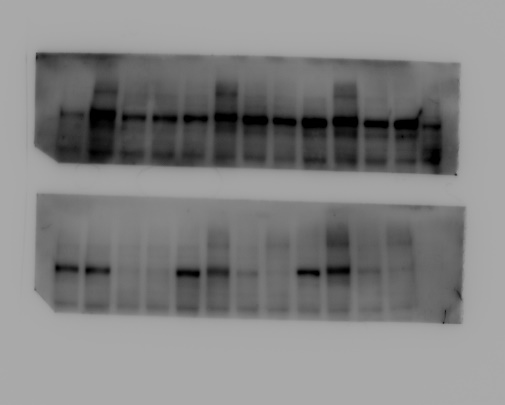

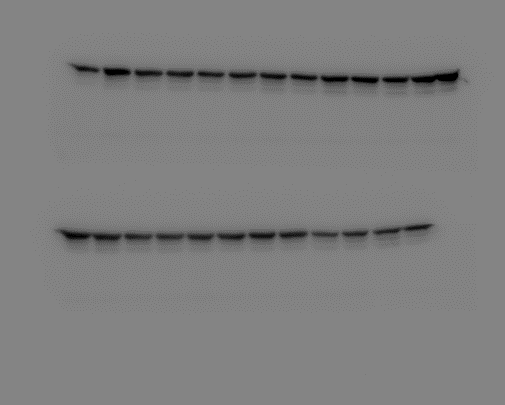


EGFR Actin

E


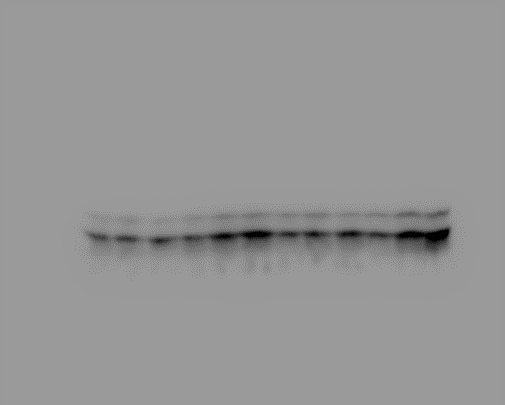

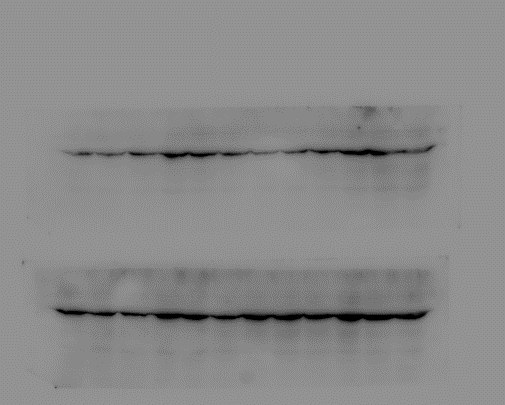


LC3 Actin

F


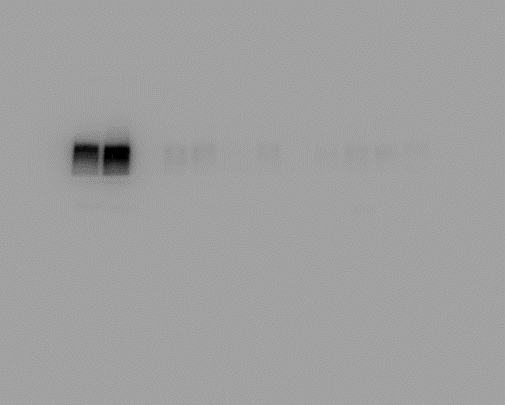

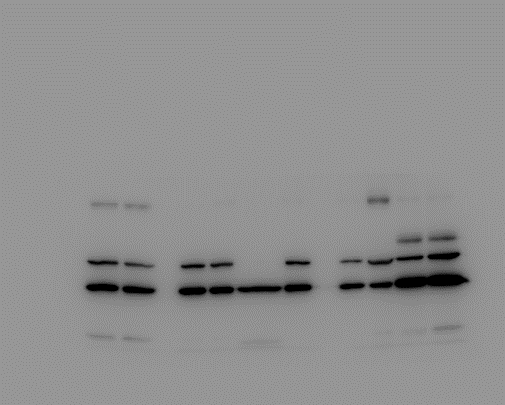


EGFR Actin

Figure 6

A


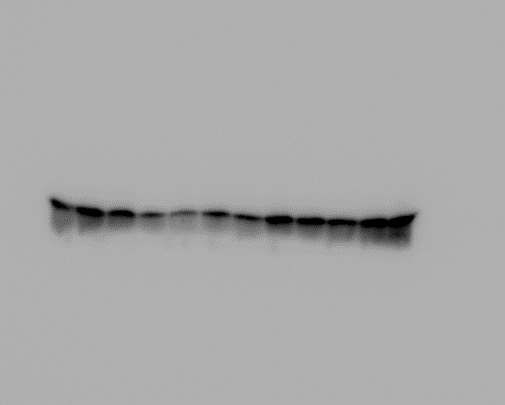

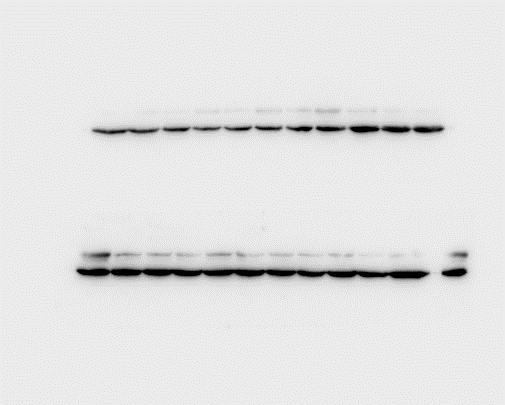


LC3 Actin

B


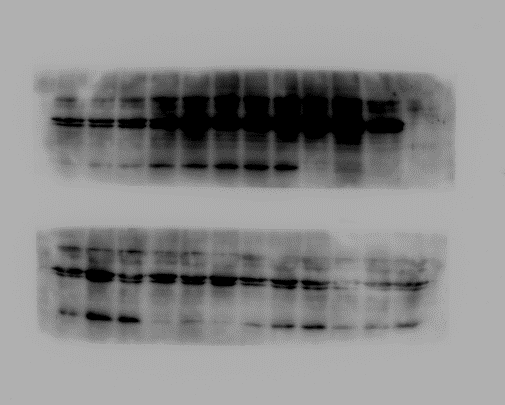
 Grb2
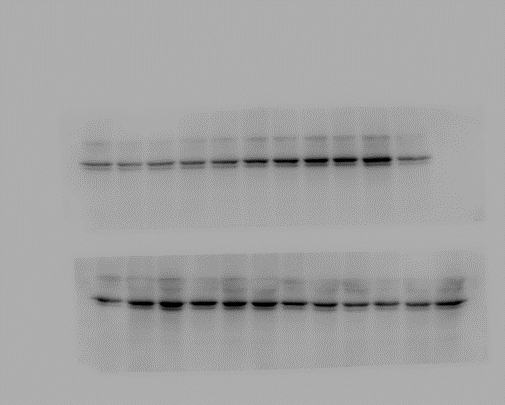
 Actin

D


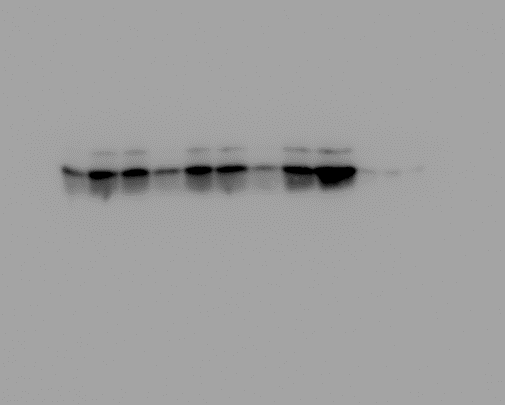

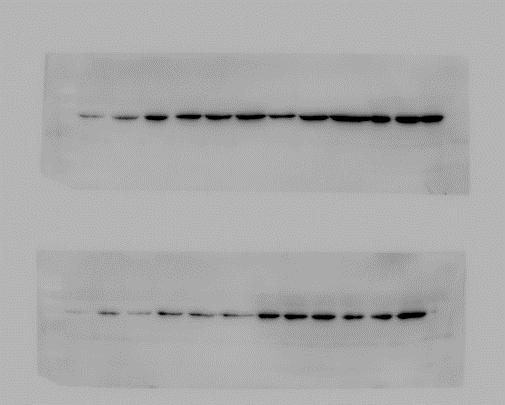


LC3 Actin

E


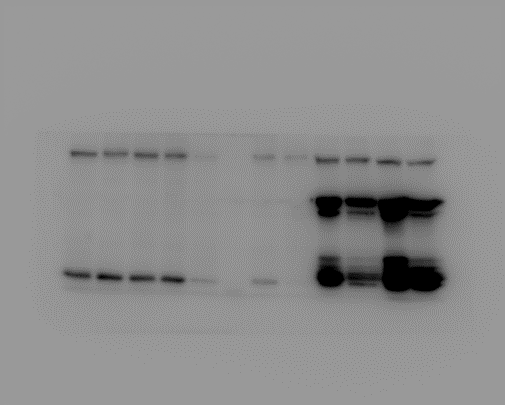

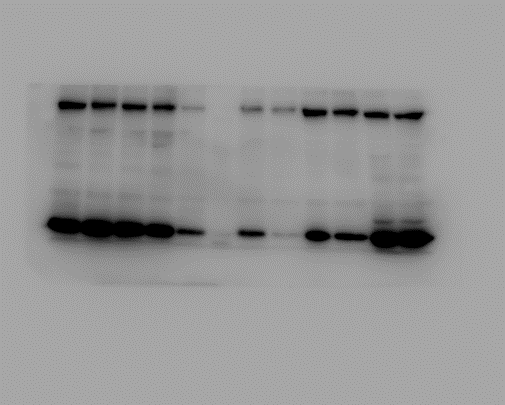


GFP Grb2

Figure 7

B


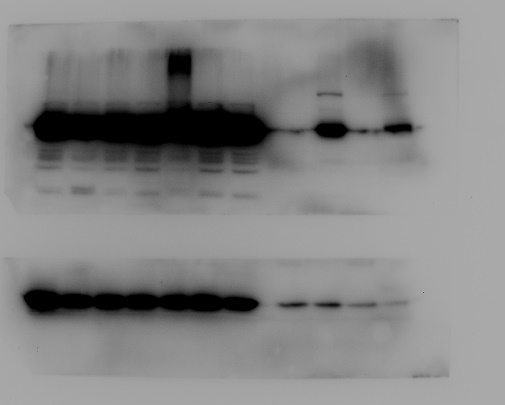

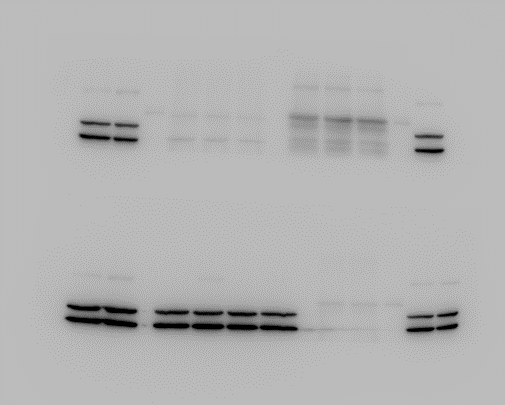


RIP1 Actin

E


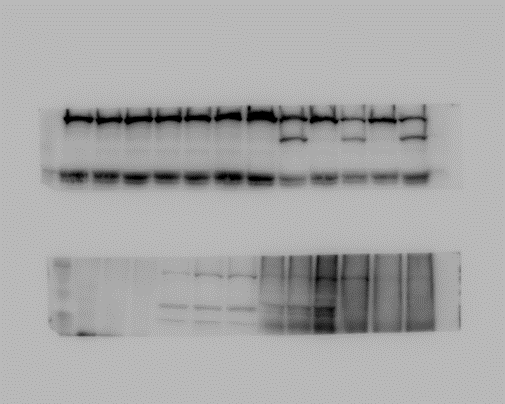

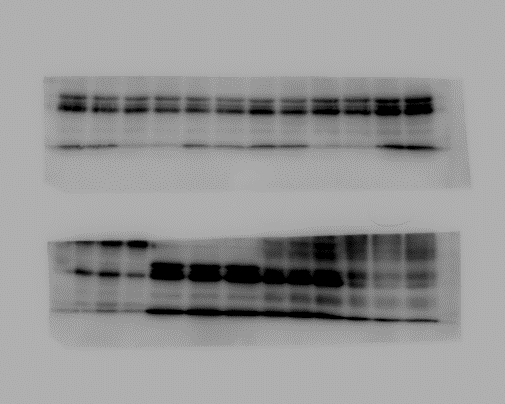


PARP Actin

**Supplemental Figure 1**

C


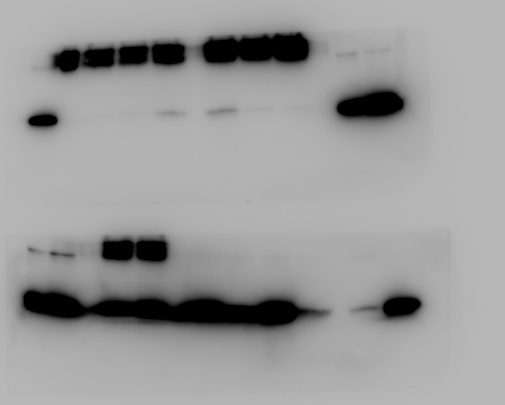

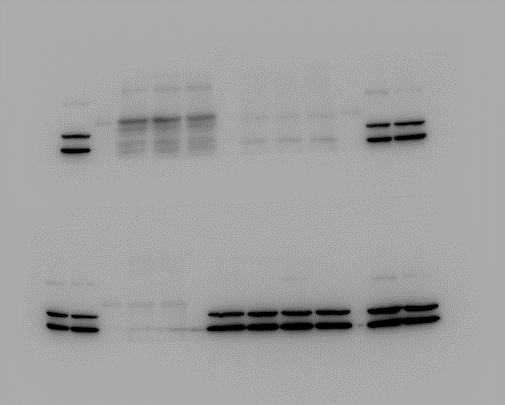


pH2AX Actin

**Supplemental Figure 2**

A


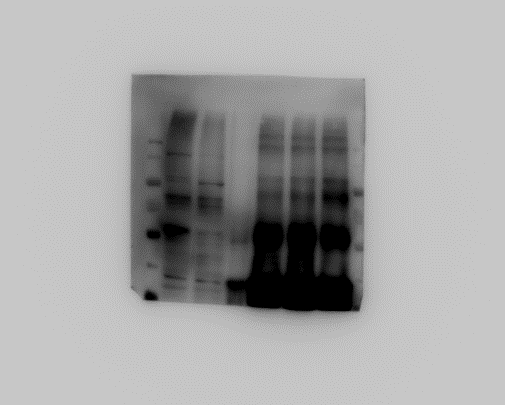

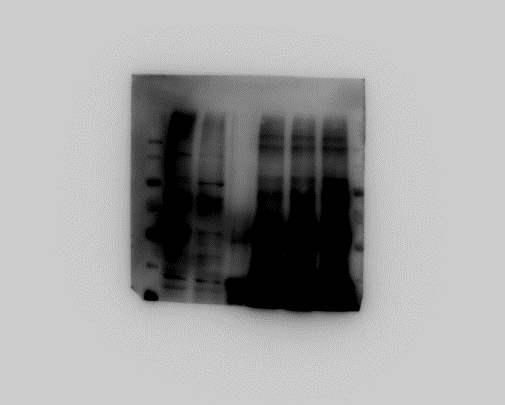


RIP1 RIP1


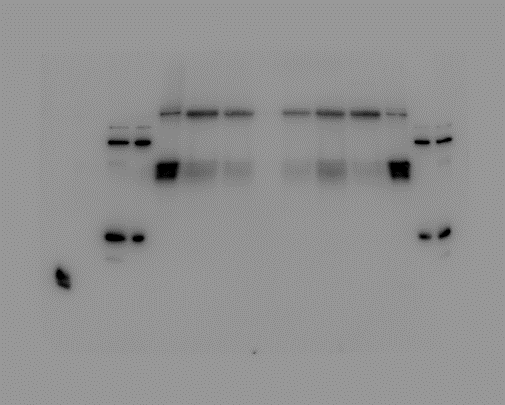
 Actin

B


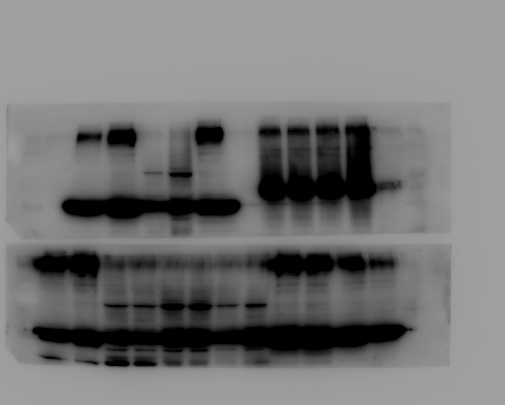

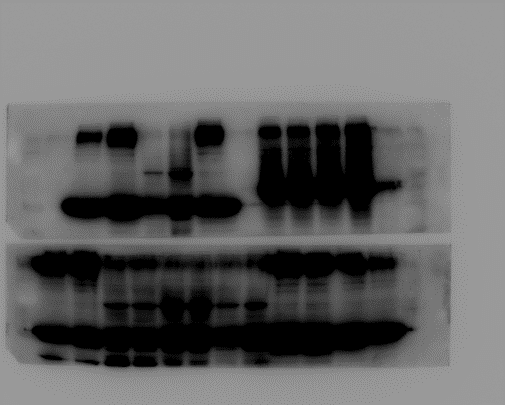


RIP1 RIP1


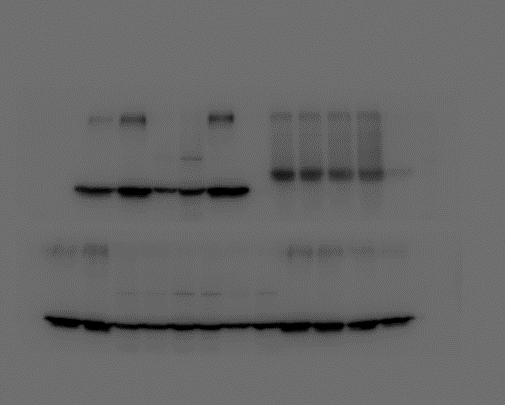


**Actin**

**C**


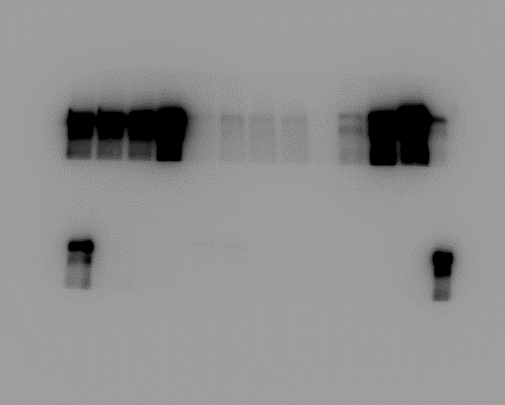

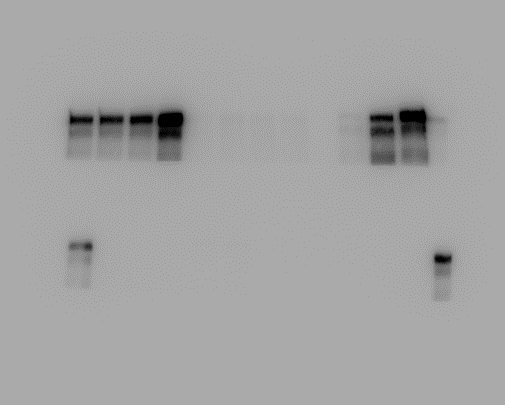


EGFR EGFR


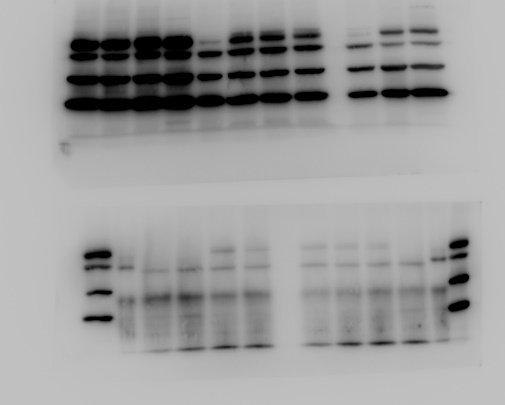


Actin
